# Adoptive transfer of ILC2s reveals tumor homing in glioblastoma: a proof-of-concept study

**DOI:** 10.3389/fonc.2026.1776061

**Published:** 2026-02-26

**Authors:** Lei P. Wang, Bidhan Bhandari, Sahar Emami Naeini, Jack C. Yu, Ali S. Arbab, Nancy Young, Évila Lopes Salles, Babak Baban

**Affiliations:** 1 Dental College of Georgia (DCG) Center for Excellence in Research, Scholarship, and Innovation (CERSI) Augusta University, Augusta, GA, United States; 2 Department of Oral Biology, Dental College of Georgia, Augusta University, Augusta GA, United States; 3 Department of Neurology, Medical College of Georgia, Augusta University, Augusta, GA, United States; 4 Biomedical Research Associates of Georgia LLC, Augusta, GA, United States; 5 Department of Surgery, Medical College of Georgia, Augusta University, Augusta, GA, United States; 6 Georgia Cancer Center, Medical College of Georgia, Augusta University, Augusta, GA, United States; 7 Department of General Dentistry, Dental College of Georgia, Augusta University, Augusta, GA, United States

**Keywords:** adaptive cell transfer, blood brain-barrier, cancer immunology, glioblastoma, immune cell trafficking, innate lymphoid cells (ILC2), tumor immune microenvironment

## Abstract

**Introduction:**

Glioblastoma (GBM) is an aggressive brain tumor with limited treatment options and poor immune cell infiltration. Although cellular immunotherapies have transformed cancer treatment, they remain largely ineffective against GBM due to the restrictive blood–brain barrier (BBB) and a profoundly immunosuppressive tumor microenvironment. Innate lymphoid cells type 2 (ILC2s) have recently emerged as potential candidates for immune-based approaches because of their regenerative and immunomodulatory functions.

**Methods:**

Bone marrow–derived ILC2s from C57BL/6 mice were fluorescently labeled and intravenously transferred into hosts bearing orthotopic, luciferase-expressing GL261 glioblastoma tumors. Immune cell localization was assessed using fluorescence imaging and flow cytometric analyses of brain, tumor tissue, meninges, and peripheral organs.

**Results:**

Systemically administered ILC2s accessed the CNS and were detected within intracranial glioblastoma tumors and meninges. Transferred ILC2s localized to tumor tissue and meninges and were also identified in peripheral organs, demonstrating effective trafficking and tumor homing in an immunocompetent model. No measurable reduction in tumor growth was observed.

**Conclusion:**

These findings establish a proof-of-concept that adoptively transferred ILC2s can access and localize within glioblastoma *in vivo*. While not associated with tumor growth inhibition in this study, the results provide foundational insight into innate immune cell trafficking to central nervous system tumors and support further investigation into the immunomodulatory potential of ILC2s in GBM.

## Introduction

The Glioblastoma (GBM) remains one of the most aggressive primary brain tumors, characterized by rapid progression, therapeutic resistance, and a profoundly immuno-suppressive microenvironment. Despite advances in surgery, radiation, and chemo-therapy, prognosis has changed little over the past decades, with a median survival of only 12–18 months (1, 2).

Immunotherapies, particularly cellular approaches such as chimeric antigen receptor (CAR) T cells, natural killer (NK) cells, and tumor-infiltrating lymphocytes, have shown encouraging results in other cancers but face major challenges in GBM (3–6). These challenges include limited trafficking of immune cells across the blood–brain barrier (BBB), potent immunosuppression within the tumor microenvironment, and pronounced intratumoral heterogeneity (5). The glioblastoma microenvironment is characterized by complex structural and vascular features that actively impede immune cell infiltration and function (7, 8). Overcoming these barriers requires innovative cellular platforms capable of entering the central nervous system (CNS) and engaging tumor tissue effectively.

Innate lymphoid cells (ILCs) represent a relatively recent addition to the immune landscape. Paralleling T cell subsets but lacking antigen specificity, ILCs function as tissue-resident sentinels that contribute to immune surveillance, tissue repair, and inflammation (9, 10). Emerging evidence has identified ILCs as potential contributors to tumor immunity; however, their roles remain incompletely defined, particularly in the context of GBM (11–14). Among these populations, group 2 innate lymphoid cells (ILC2s) are of particular interest due to their involvement in tissue repair, immune modulation, and cross-talk with both innate and adaptive immune compartments (15–17). Whether ILC2s can infiltrate CNS tumors and persist within the glioblastoma microenvironment has not been experimentally demonstrated.

Recent reports suggesting that ILCs can migrate beyond classical barrier tissues raise the possibility that ILC2s may access intracranial tumors (13, 18). Here, we report the first adoptive transfer of bone marrow–derived ILC2s into a mouse model of orthotopic GBM and track their migration using fluorescent labeling.

Our findings provide experimental proof-of-concept that ILC2s can access and localize within CNS tumors. While not intended to demonstrate therapeutic efficacy, this work establishes a biological foundation for future studies investigating the mechanisms, functional consequences, and translational potential of ILC2 interactions within the glioblastoma microenvironment.

## Materials and methods

### Animals and ethics

Wild-type male C57BL/6 mice (10–12 weeks old; n = 6 from three independent cohorts; Jackson Laboratories, Bar Harbor, ME) were used. Animals were maintained under standard housing conditions with ad libitum access to food and water. All procedures were approved by the Augusta University Institutional Animal Care and Use Committee [IACUC #2011-0062] and conducted in accordance with the NIH Guide for the Care and Use of Laboratory Animals. The study was adhered to the ARRIVE guidelines for re-porting animal research (ARRIVE 2.0 Essential 10 guidelines).

### Orthotopic GBM model

The luciferase-expressing GL261 line (GL261-Luc2) used in this study was obtained from the Georgia Cancer Center Cell Repository. This line is derived from the parental GL261 glioma cell line (NCI/ATCC origin) and stably transduced with firefly luciferase, allowing reliable *in vivo* bioluminescence imaging. The GL261 orthotopic model was selected as it represents an immunocompetent, syngeneic system that recapitulates key features of human glioblastoma, including an immunosuppressive microenvironment and limited immune cell infiltration, while allowing investigation of immune cell trafficking without confounding allograft rejection. GL261-Luc2 cells were cultured under standard conditions and stereotactically implanted into the right striatum (3 × 10^4^ cells in 3 µL PBS) of C57BL/6 mice as described previously (1). Mice were anesthetized using 3% isoflurane for induction and maintained with 1.5–2% isoflurane throughout surgical procedures. Tumor establishment and progression were verified by *in vivo* bioluminescence imaging (IVIS Spectrum). At the endpoint, animals were euthanized by CO_2_ inhalation followed by cervical dislocation, in accordance with the American Vet-erinary Medical Association (AVMA) Guidelines for the Euthanasia of Animals.

### Isolation and adoptive transfer of bone marrow-derived ILC2s

Bone marrow cells were harvested from donor C57BL/6 mice as described previously (19). Lineage-negative (Lin^-^) ILC2s were isolated using magnetic negative selection (BioLegend antibodies against CD3ϵ, CD5, CD19, LY6G, NK1.1, CD11b, CD11c, and Ter119) followed by Streptavidin MicroBeads (Miltenyi Biotec). Purified ILC2s were ex-panded for six days in RPMI-1640 medium supplemented with 10 ng/mL recombinant IL-7 and IL-33 (R&D Systems). ILC2 identity was confirmed by flow cytometry (Lin^-^CD45^+^Sca-1^+^ST2^+^GATA3^+^CD25^+^ICOS^+^c-Kit^+^IL-5^+^IL-13^+^). Prior to adoptive transfer, activated ILC2s were labeled with 5 µM carboxyfluorescein succinimidyl ester (CFSE; Invitrogen) for 10 minutes at 37 °C, following established protocols (18–20). CFSE labeling provides a stable, heritable fluorescent marker that allows definitive tracking of trans-ferred cells in recipient animals without requiring re-staining for phenotypic markers (20–22). To eliminate the possibility of free CFSE contributing to tissue fluorescence, la-beled ILC2s were washed three times with complete medium until the supernatant ex-hibited no measurable fluorescence. CFSE is an amine-reactive, intracellularly retained dye and does not dissociate once bound. Labeled ILC2s were intravenously injected via the tail vein (3 × 10^6^ cells/mouse) on day 9 post-tumor implantation, a time point ensuring established tumor growth. The cell dose was adapted from established ILC2 adoptive transfer protocols (23) and adjusted to ensure sufficient cell numbers for reliable detection and tracking in the CNS while avoiding potential adverse effects from excessive cell infusion. Control tumor-bearing mice received PBS injections. In flow cytometric analysis of recipient tissues, CD45^+^CFSE^+^ events represent transferred ILC2s, as CFSE was applied exclusively to the donor population. During downstream analyses, CFSE^+^ events were only considered ILC2s if they also expressed the expected immune phenotype (Lin^-^CD45^+^Sca-1^+^ST2^+^GATA3^+^). No CFSE^+^ events were detected in the brains of PBS-injected control mice, confirming signal specificity. This methodology is consistent with standard practice in adoptive immune cell transfer studies (18–20).

### Imaging and flow cytometry

Bioluminescence imaging confirmed tumor growth, while fluorescence microscopy visualized CFSE^+^ ILC2s in the brain, meninges and peripheral organs (spleen, lung). Single-cell suspensions from these tissues were analyzed for CFSE^+^ ILC2s and other ILC subsets using a NovoCyte Quanteon flow cytometer (Agilent Technologies). Data were processed with FlowJo software (V10) as described previously (8). Fluorescence quanti-fication was performed as mean pixel intensity (MFI) per microscope field. For each mouse, five non-overlapping regions were imaged at identical exposure settings, and the CFSE fluorescence channel was extracted and background-subtracted using ImageJ. The average pixel intensity per field per animal was used for statistical comparisons.

### Bioluminescence quantification

Tumor-associated photon emission was quantified using Living Image 4.7 software. For each mouse, a fixed-size region of interest (ROI) was drawn over the tumor, and an identical ROI was placed over a non-tumor brain region to obtain background signal. Background-subtracted radiance (photons/sec) was used for all comparisons. All imaging sessions were performed using identical exposure settings. Tumor-bearing mice included n = 6 per condition, derived from three independent experimental cohorts.

### Statistical analysis

All data were analyzed using GraphPad Prism (version 9). Frequencies of CFSE^+^ ILC2s in brain, meninges, spleen, and lung were compared using one-way ANOVA followed by Tukey’s *post-hoc* test. Quantification of CFSE fluorescence intensity in tissue sections was based on mean pixel intensity per field and analyzed using one-way ANOVA. For tumor bioluminescence, background-subtracted radiance values were compared between groups using unpaired t-tests, whereas longitudinal comparisons within the same animals used paired t-tests. All values are reported as mean ± SEM, and statistical significance was defined as p < 0.05.

## Results

### ILC2s traffic to and infiltrate brain tumors following adoptive transfer

**Figure 1:**
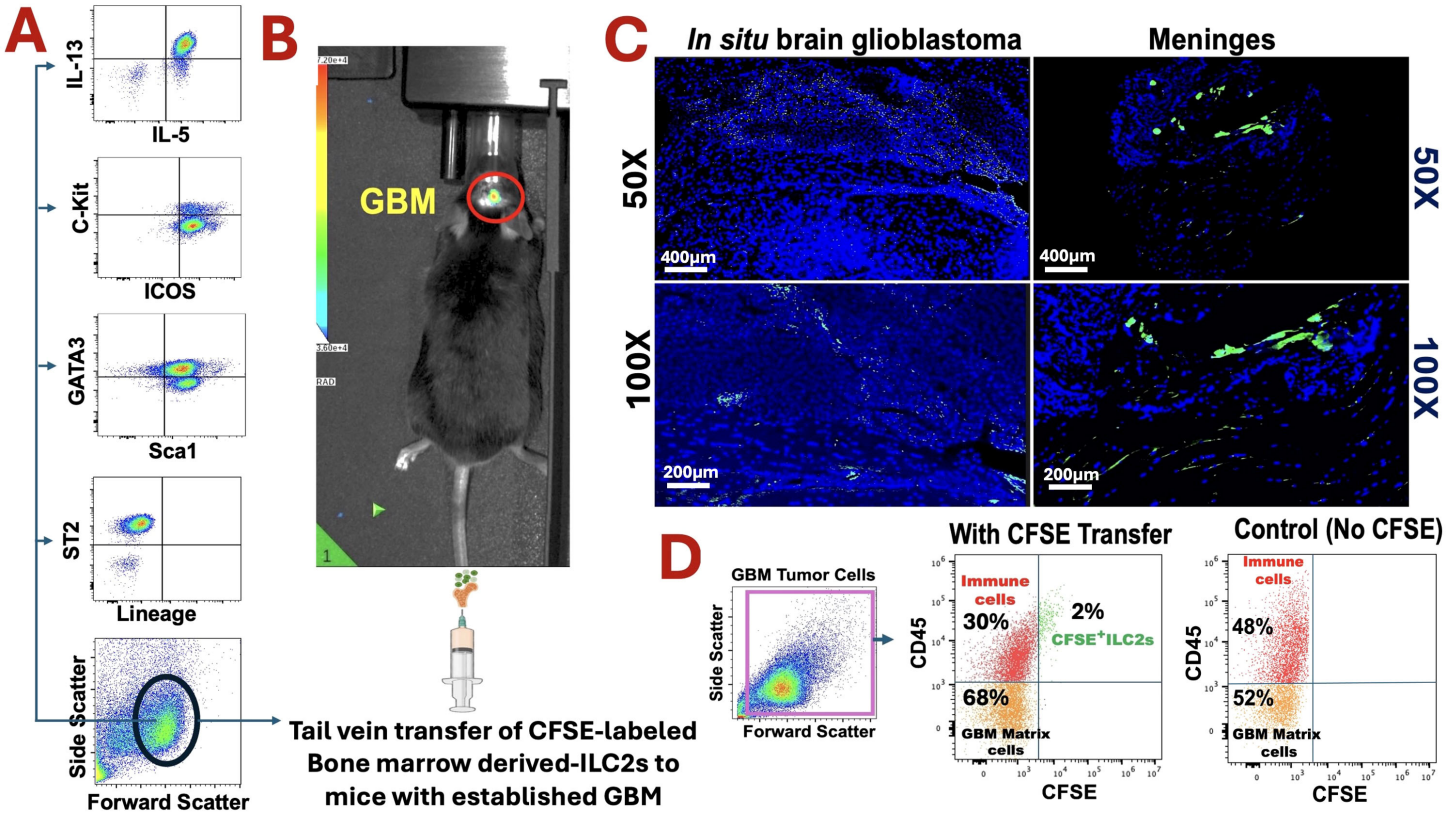
Adoptively transferred bone marrow–derived ILC2s traffic to glioblastoma following systemic administration. **(A)** Phenotypic Flow cytometric characterization of bone marrow–derived ILC2s prior to transfer, confirming a canonical ILC2 phenotype based on cytokine expression and surface markers. **(B)** Experimental workflow and *in vivo* bioluminescence imaging confirming establishment of orthotopic GL261-luciferase glioblastoma before intravenous transfer of CFSE-labeled ILC2s. **(C)** Representative fluorescence microscopy images obtained 96 hours after transfer showing CFSE-labeled ILC2s within glioblastoma tissue and meninges at indicated magnifications; nuclei are counterstained with DAPI. **(D)** Flow cytometric identification of transferred CD45^+^CFSE^+^ ILC2s within tumor-associated immune populations. Control animals receiving no CFSE-labeled cells show no detectable CFSE signal.

Bioluminescence imaging confirmed reliable establishment of orthotopic GBM tumors by day 9 post-implantation, providing a stable model for subsequent ILC2 transfer (**Figure 1A**). At this stage, CFSE-labeled bone marrow–derived ILC2s were intravenously administered to tumor-bearing mice to assess their migratory behavior and CNS entry.

At 96 hours post-transfer, fluorescence imaging revealed distinct CFSE^+^ signals within the meninges, brain, and tumor parenchyma (**Figure 1B**). Flow cytometric analysis confirmed that these infiltrating cells retained canonical ILC2 markers (Lin^-^CD45^+^Sca-1^+^ST2^+^GATA3^+^), identifying them as a discrete population within the glioblastoma microenvironment (**Figure 1C**). This represents, to our knowledge, the first direct indication that adoptively transferred ILC2s can access the CNS and localize within glioblastoma tissue following systemic delivery. While these findings demonstrate CNS entry, the precise route of infiltration, whether via direct BBB crossing, meningeal pathways, or perivascular access, cannot be definitively determined from the current data. To ensure that the CFSE^+^ signals represented intact ILC2s rather than free dye, we implemented multiple verification steps. CFSE binds covalently to intracellular amines and does not dissociate once incorporated; labeled ILC2s were therefore washed repeatedly until no detectable fluorescence remained in the supernatant. The CFSE^+^ events identified in brain and tumor tissue also co-expressed canonical ILC2 markers (Lin^-^CD45^+^Sca-1^+^ST2^+^GATA3^+^), a phenotype that cannot arise from free dye (**Figure 1D**). In tissue sections, CFSE^+^ structures displayed discrete, nucleated, cell-like morphology rather than diffuse extracellular staining, and no CFSE signal was observed in PBS-injected tumor-bearing controls. Together, these findings support that the CFSE^+^ signals reliably reflect transferred ILC2s.

### Systemic and CNS-associated distribution of adoptively transferred ILC2s

**Figure 2:**
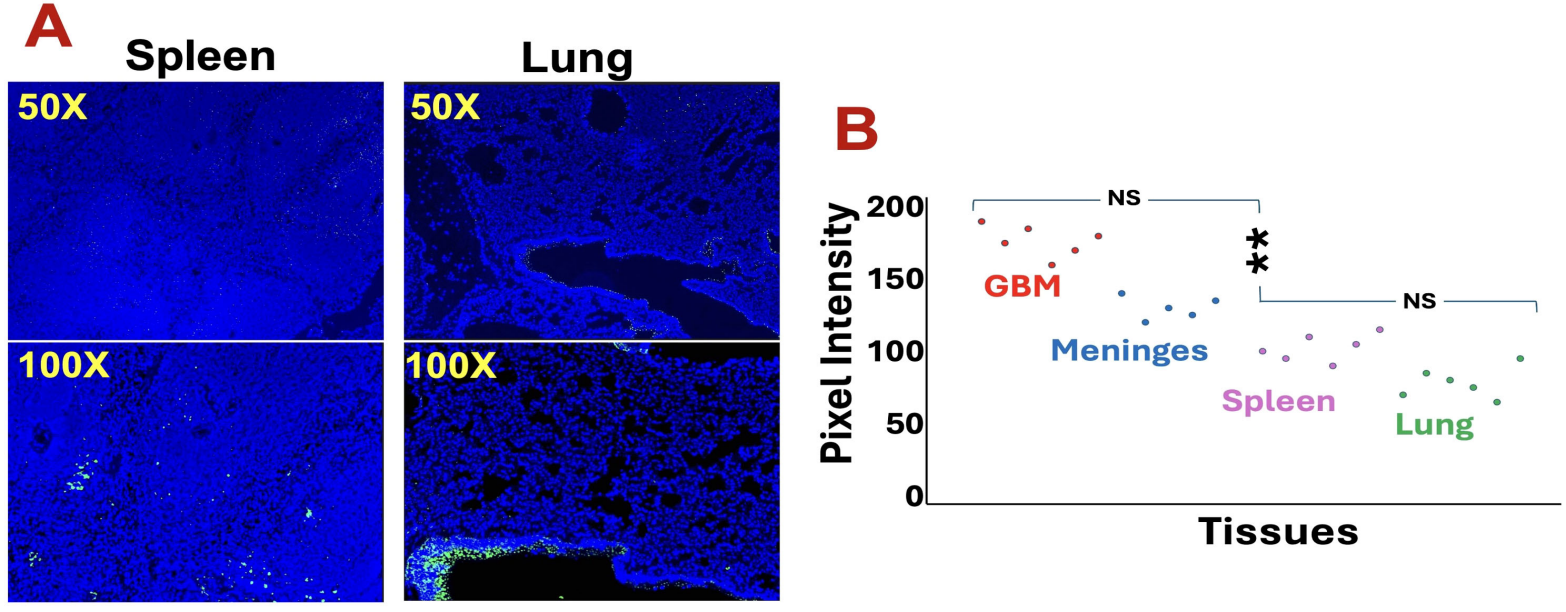
Adoptively transferred ILC2s exhibit both systemic and CNS-associated distribution. **(A)** Representative fluorescence images showing CFSE^+^ ILC2s (green) detected in spleen and lung tissue 96 hours after intravenous transfer. Nuclei are counterstained with DAPI (blue). Images are shown at 50× and 100× magnifications. **(B)** Quantitative comparison of CFSE fluorescence intensity across tissues demonstrating higher ILC2 accumulation in glioblastoma (GBM) and meninges compared with peripheral organs. Data represent mean ± SEM (n = 6 mice, three independent experiments). p < 0.01 (**), NS, not significant.

Beyond the brain, CFSE^+^ ILC2s were also detected in peripheral tissues, including spleen and lung (**Figure 2A**), suggesting preserved systemic migratory potential. However, their frequency was markedly higher in the meninges and tumor regions (**Figure 2B**), suggesting a degree of preferential accumulation within CNS compartments. These findings indicate that ex vivo–expanded ILC2s maintain the capacity to traffic through the circulation and traverse the blood–brain barrier under the inflammatory conditions associated with GBM.

### Unmodified ILC2s infiltrate but do not alter glioblastoma growth

**Figure 3:**
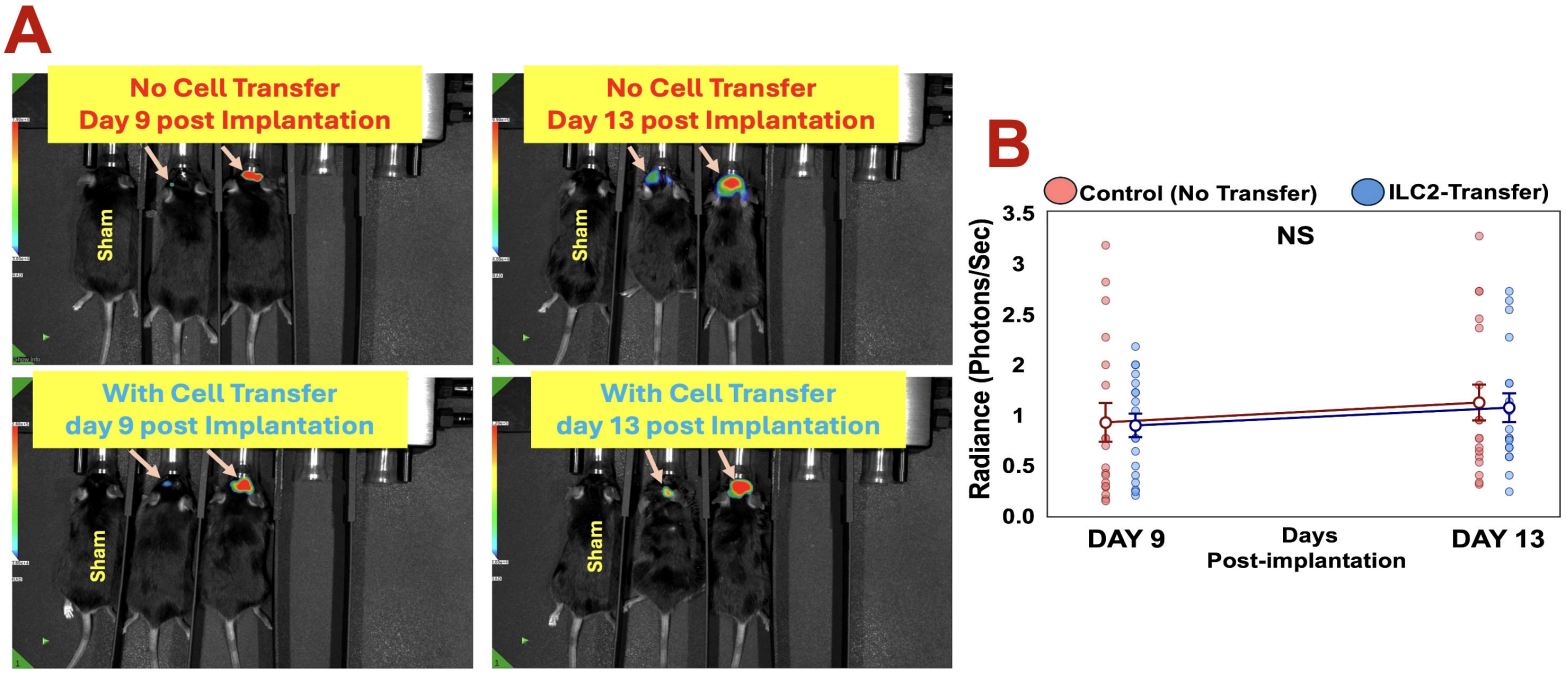
Adoptive transfer of unmodified ILC2s does not alter glioblastoma growth. **(A)** Representative *in vivo* bioluminescence images of GL261-luciferase tumor–bearing mice without (top) or with (bottom) intravenous ILC2 transfer at days 9 and 13 post-implantation; sham-operated mice are shown as controls. **(B)** Individual tumor radiance measurements at days 9 and 13 post-implantation in control and ILC2-treated mice. Symbols represent individual animals, with mean ± SEM indicated. No significant differences in tumor radiance were observed between groups at either time point (NS).

Despite successful homing and infiltration, ILC2 transfer did not significantly alter tumor progression as determined by longitudinal bioluminescence imaging (**Figure 3A**). Individual tumor radiance measurements at days 9 and 13 post-implantation showed comparable growth trajectories between ILC2-treated and control mice (**Figure 3B**), with no significant difference in tumor burden at either timepoint. These findings suggest that unmodified ILC2s alone are insufficient to exert antitumor activity within the GBM microenvironment. Nevertheless, their observed ability to enter and persist within intracranial tumors support a proof-of-concept for using ILC2s as a deliverable cell platform. Future efforts will focus on enhancing their therapeutic potential through cytokine priming, checkpoint modulation, or genetic engineering to improve tumor specificity and efficacy.

## Discussion

This study provides the first experimental evidence that bone marrow–derived ILC2s can traffic to and localize within glioblastoma tissue following systemic administration. Although adoptive transfer did not result in measurable changes in tumor growth, the demonstration that ILC2s can access the CNS and persist within the glioblastoma microenvironment represents an important conceptual advance for understanding immune cell accessibility to central nervous system tumors.

ILC2s are increasingly recognized for their regenerative and immunomodulatory roles in peripheral tissues and solid tumors. Through the production of type 2 cytokines and interactions with myeloid and adaptive immune cells, ILC2s can influence macrophage polarization, tissue remodeling, and immune homeostasis. Establishing that systemically delivered ILC2s can access and localize within the glioblastoma niche provides a biological foundation for investigating how these cells may interact with the tumor microenvironment and contribute to immune modulation in the brain. The observed ILC2 trafficking and tumor localization can be contextualized within the broader landscape of immune cell infiltration in GBM. Adoptively transferred T cells, particularly CAR-T cells, have demonstrated variable CNS access in preclinical models, with efficacy often limited by insufficient tumor infiltration, T cell exhaustion, and immunosuppressive signals within the glioblastoma microenvironment (3–5). Similarly, NK cell therapies have shown promise in hematologic malignancies but face challenges in solid tumors, including limited persistence and reduced cytotoxicity in hypoxic, immunosuppressive conditions (3–5). The capacity of systemically administered ILC2s to localize within both tumor parenchyma and meninges suggests migratory behavior that may differ from conventional adaptive immune cells, potentially reflecting innate trafficking mechanisms responsive to tissue damage signals or inflammatory cues. Whether ILC2s exhibit advantages in CNS access compared to T or NK cells, or whether their trafficking is similarly constrained by the glioblastoma microenvironment, remains an open question that warrants direct comparative investigation in future studies.

As a proof-of-concept study, several limitations should be acknowledged. The post-transfer observation window was relatively short (96 hours), limiting assessment of long-term persistence, functional adaptation, or downstream immunological effects of transferred ILC2s. In addition, the cells were not preconditioned or engineered to enhance tumor specificity or effector function. Future studies extending the observation period and incorporating targeted strategies, such as cytokine priming, receptor engineering, or combination approaches involving IL-33 or TGF-β pathway modulation, will be necessary to define the durability and functional relevance of ILC2 tumor infiltration.

Beyond its immediate findings, this work establishes a practical experimental framework for studying innate lymphoid cell behavior within intracranial tumors. The ability to track ILC2 trafficking and localization *in vivo* enables future investigations into the molecular cues guiding their migration, retention, and interaction with the glioblastoma microenvironment.

Overall, these findings support the feasibility of ILC2 CNS access and brain tumor homing, providing a first proof-of-concept for adoptive ILC2 transfer as a platform to study immune cell trafficking and immunomodulatory potential in glioblastoma and other CNS malignancies. While not indicative of therapeutic efficacy at this stage, the results offer foundational insight that may inform future mechanistic and translational studies aimed at harnessing innate immune cells for brain-directed immunomodulation.

The molecular mechanisms governing ILC2 entry into the CNS remain undefined but may involve several plausible pathways. ILC2s express chemokine receptors including CXCR6 and CCR4 (24, 25), which could respond to inflammatory chemokines upregulated in the glioblastoma microenvironment (26). Additionally, integrins and adhesion molecules on ILC2s may facilitate interactions with activated endothelium or enable trafficking through perivascular spaces. The presence of ILC2s in both meninges and tumor parenchyma raises the possibility of stepwise infiltration via meningeal routes before accessing deeper brain structures, similar to patterns observed with other immune cell subsets in neuroinflammatory conditions. Whether ILC2 infiltration is driven primarily by tumor-derived inflammatory signals, BBB disruption associated with tumor growth, or innate tissue-homing properties remains to be determined and warrants investigation in future mechanistic studies.

### Limitations

As a proof-of-concept study, several limitations should be acknowledged. The sample size (n = 6 mice from three independent cohorts) established biological feasibility but limits definitive conclusions regarding ILC2 frequency, persistence, or functional heterogeneity within the tumor microenvironment. The short post-transfer observation window (96 hours) restricted assessment of long-term persistence and downstream immunological consequences.

A critical gap is the absence of functional readouts. We did not measure *in situ* cytokine production (IL-5, IL-13), evaluate effects on tumor-associated immune populations, or assess macrophage polarization. While transferred cells retained canonical ILC2 markers, whether they remained functionally competent, capable of cytokine secretion, immune cell recruitment, or tissue remodeling, was not determined. Additionally, ILC2s are functionally heterogeneous and can exhibit plasticity, with subsets potentially promoting immunosuppression (via type 2 cytokines and M2 polarization) or supporting antitumor immunity. The absence of subset-specific characterization limits understanding of whether transferred ILC2s maintained uniform phenotypes or underwent functional diversification within the tumor.

The transferred ILC2s were unmodified, likely contributing to the absence of antitumor activity. We used a single syngeneic model (GL261); generalizability to other glioblastoma models or human GBM remains undetermined. The molecular mechanisms governing ILC2 CNS entry and tumor interactions remain undefined. These limitations underscore the exploratory nature of this work and highlight critical areas for future investigation employing single-cell approaches and functional profiling to delineate ILC2 phenotypes and therapeutic potential.

## Data Availability

The original contributions presented in the study are included in the article/supplementary material. Further inquiries can be directed to the corresponding authors.
